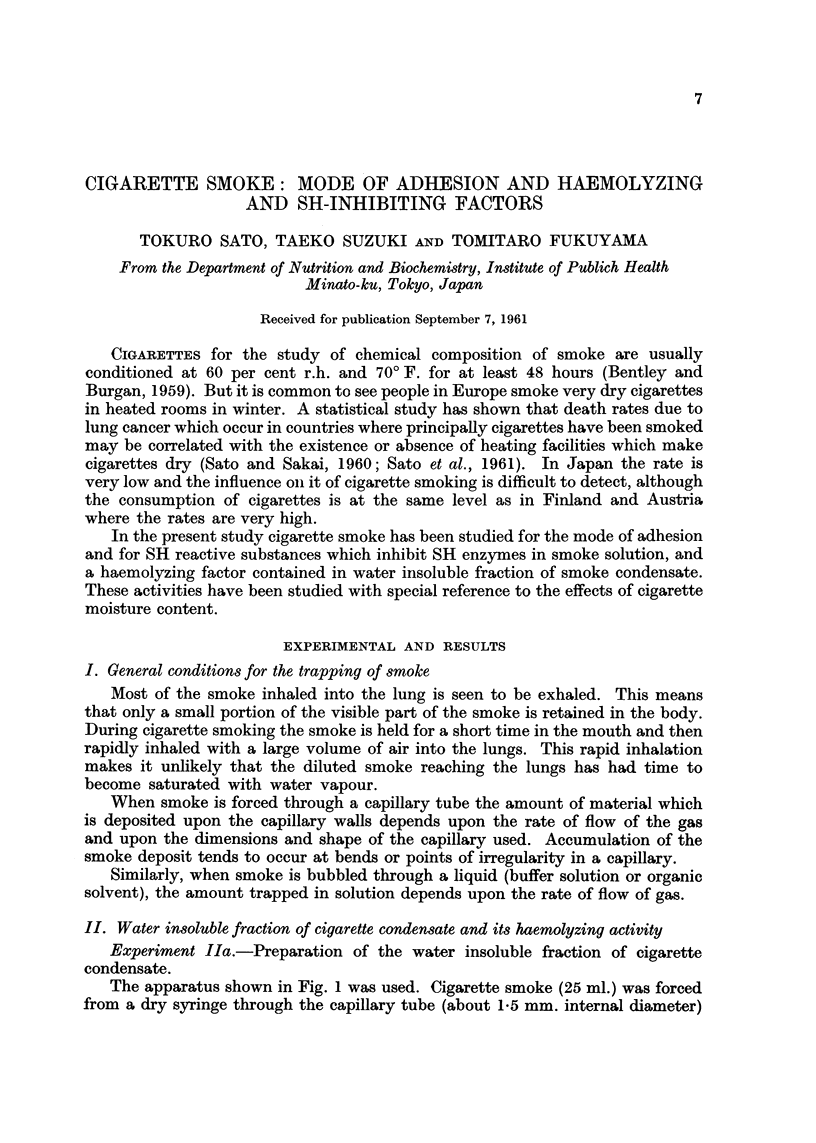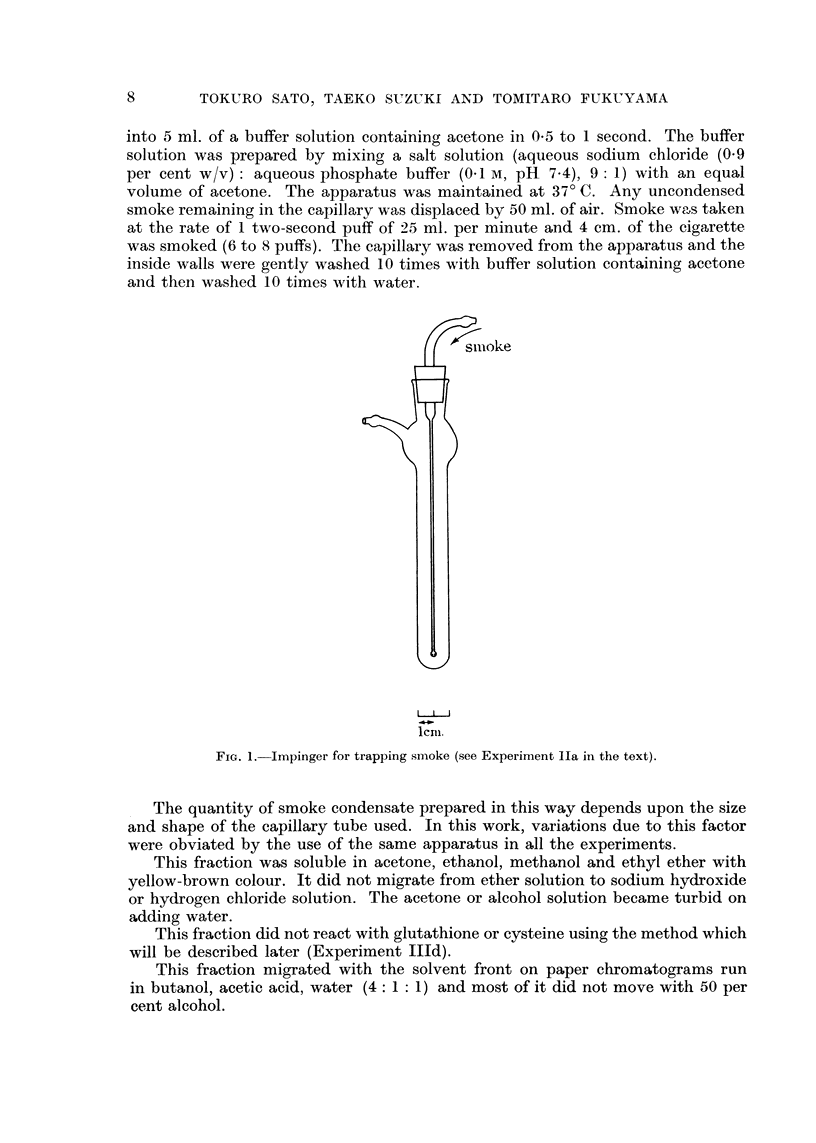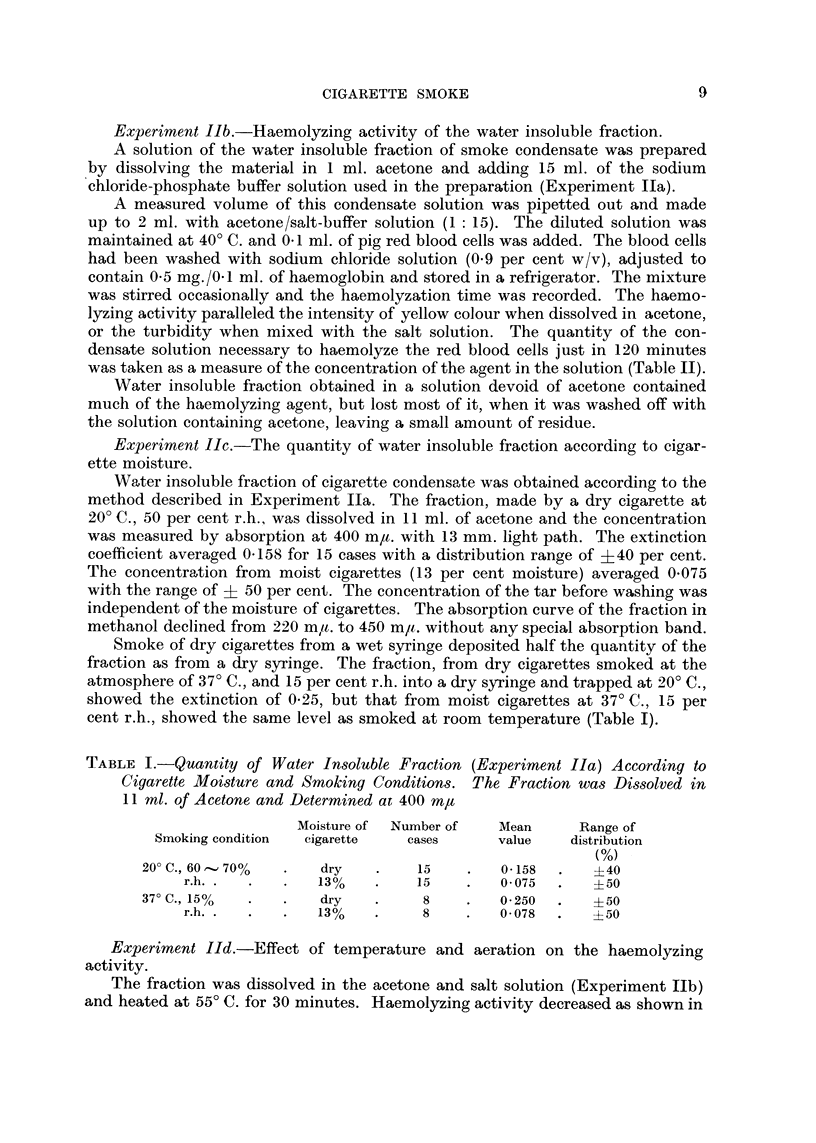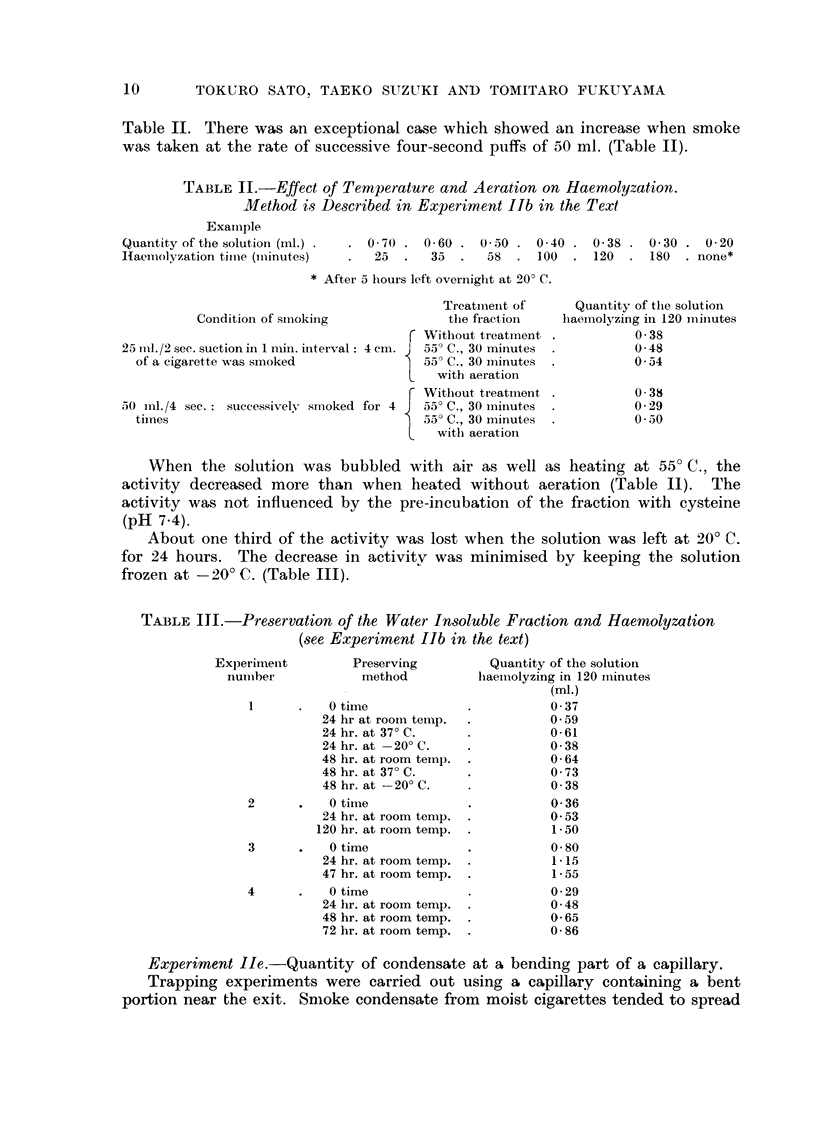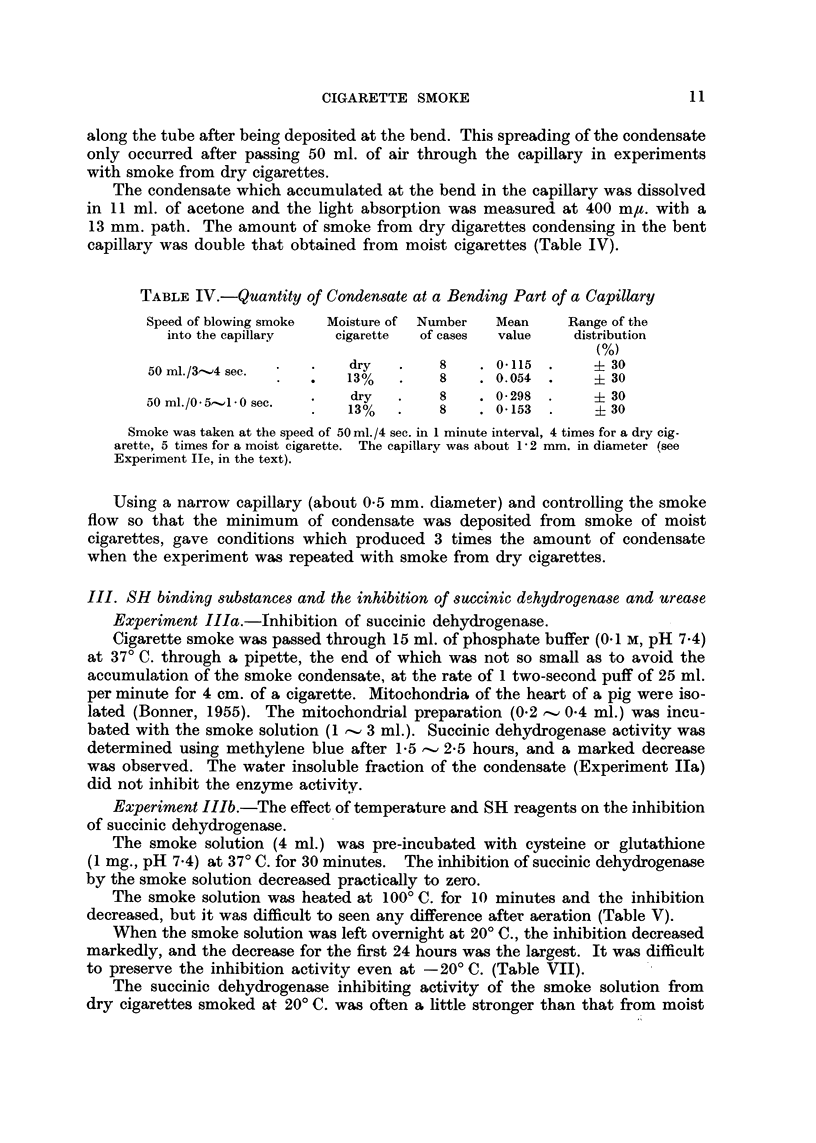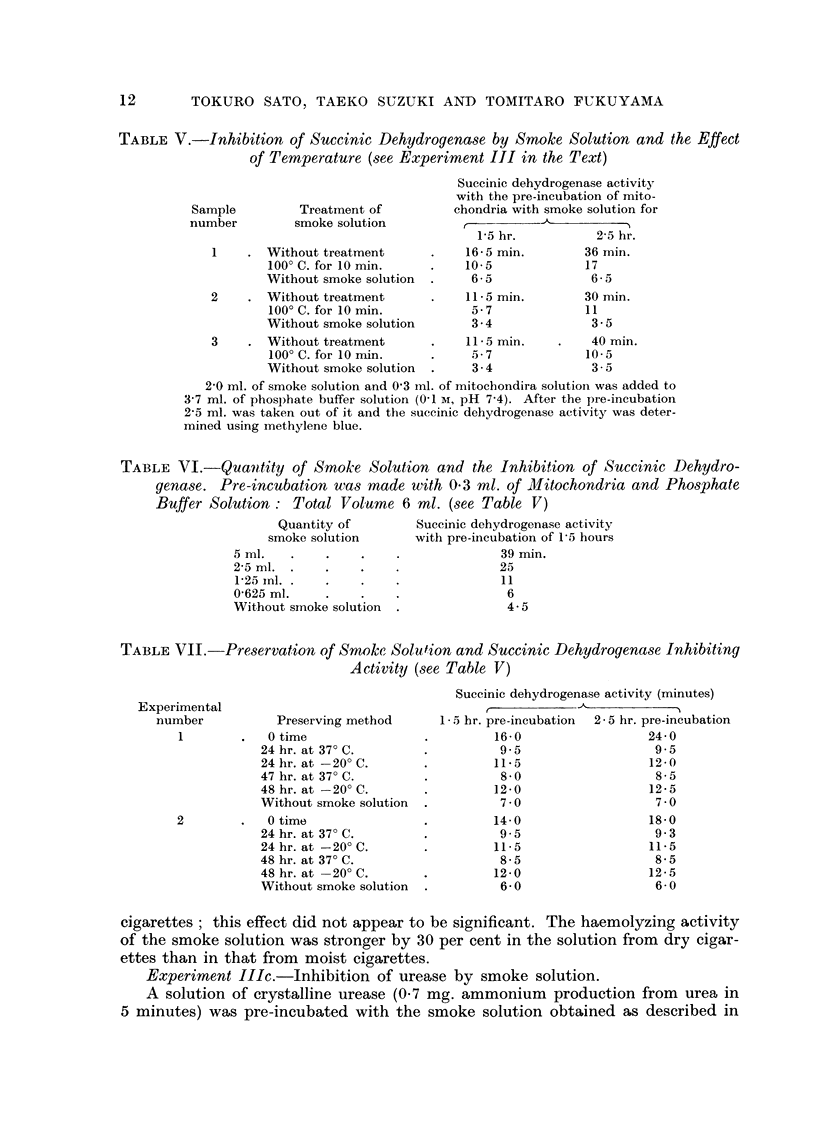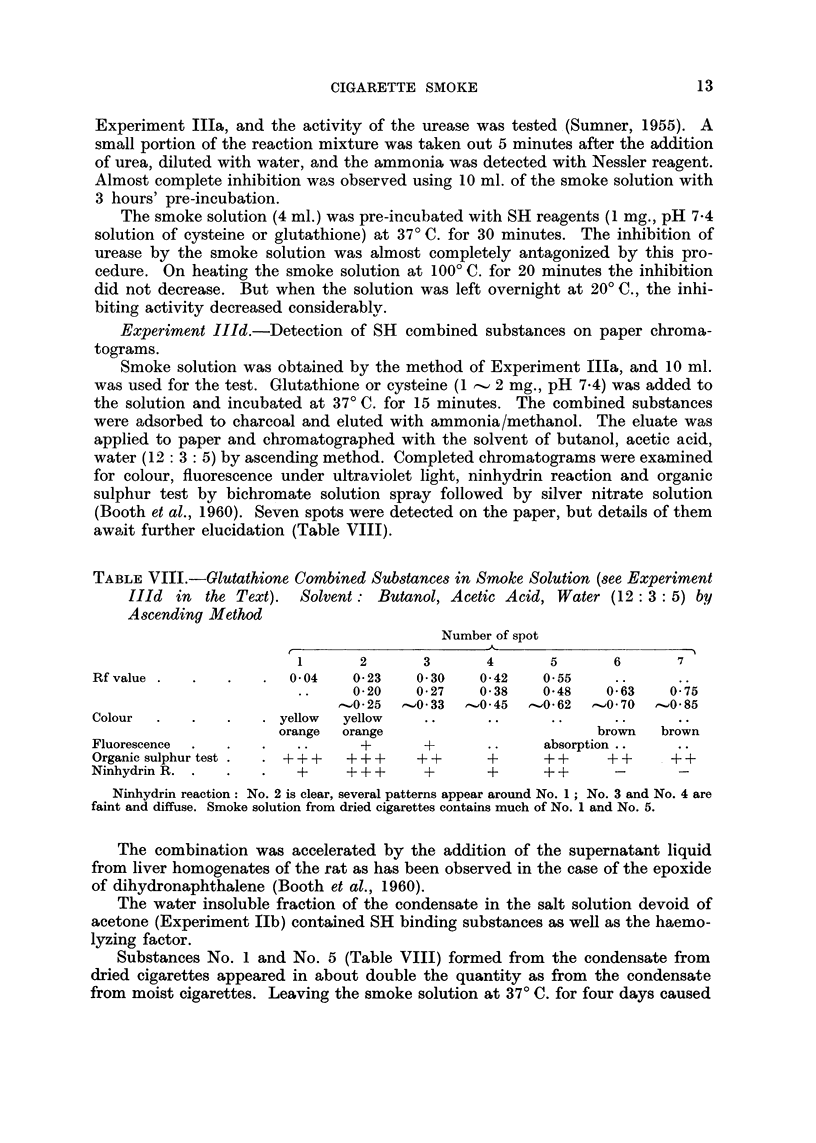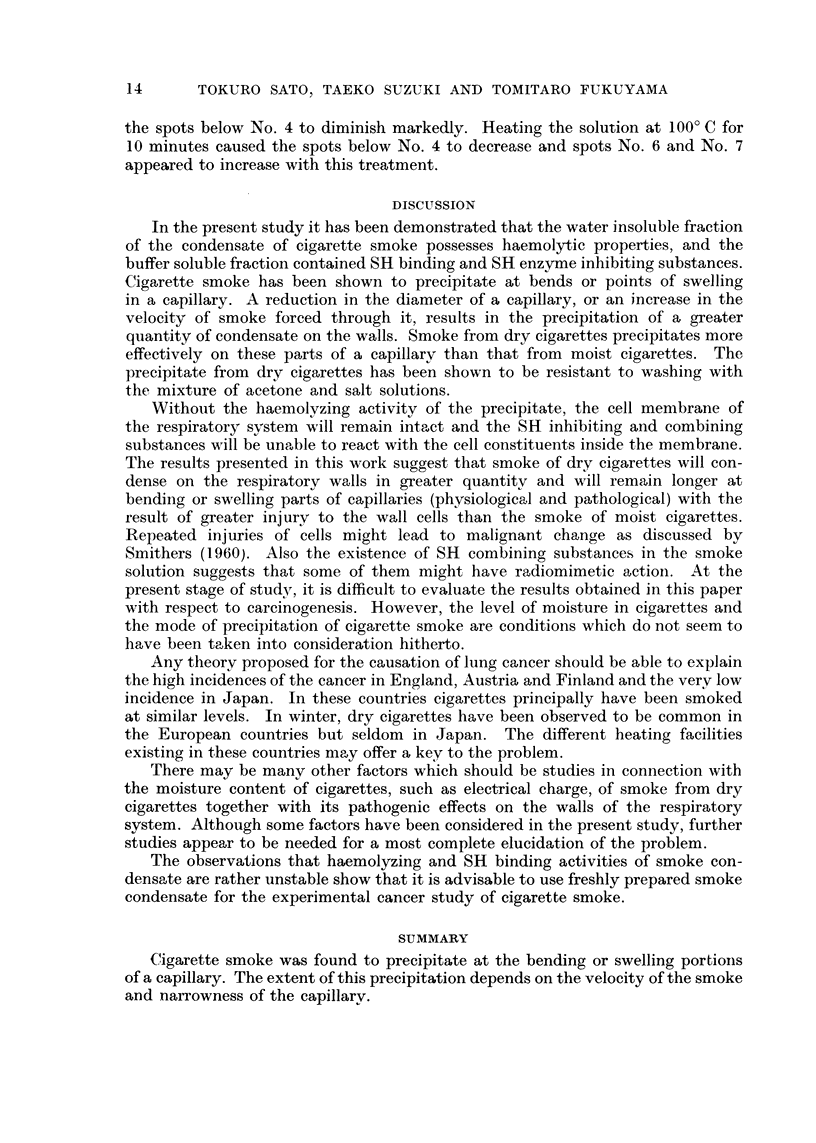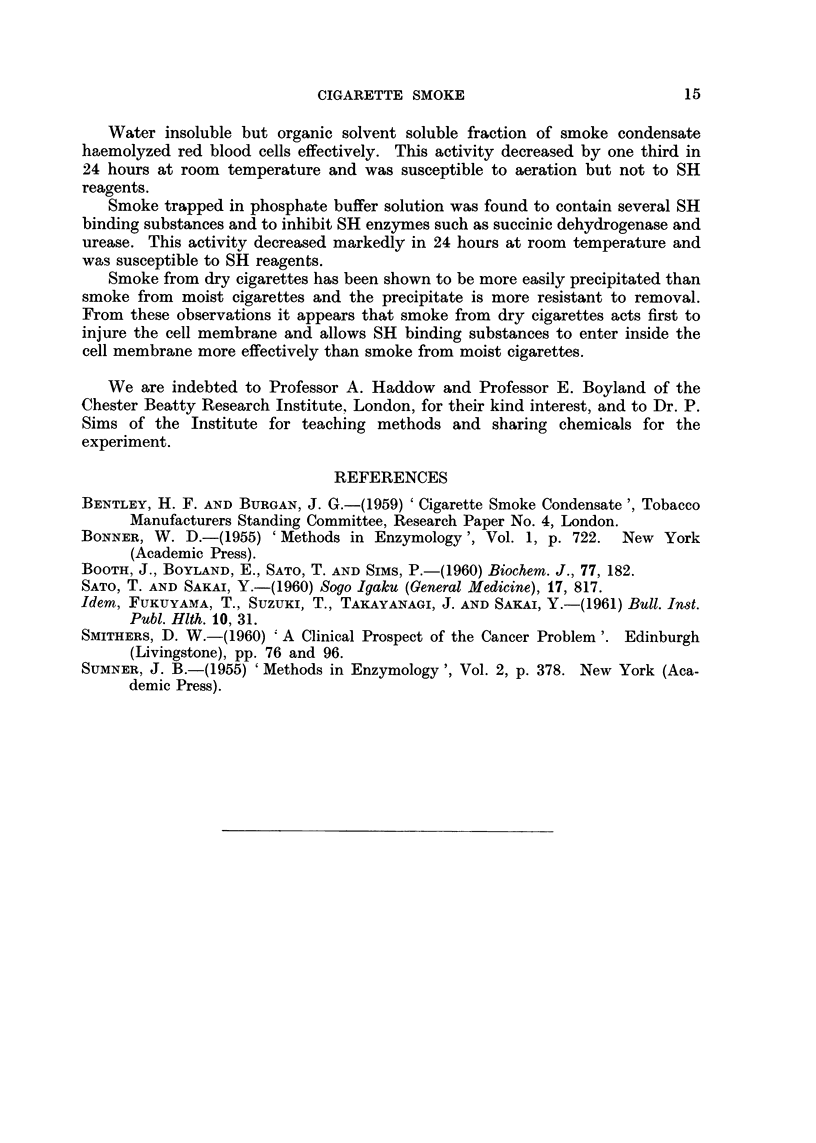# Cigarette Smoke: Mode of Adhesion and Haemolyzing and SH-inhibiting Factors

**DOI:** 10.1038/bjc.1962.2

**Published:** 1962-03

**Authors:** Tokuro Sato, Taeko Suzuki, Tomitaro Fukuyama


### CIGARETTE SMOKE: MODE OF ADHESION AND HAEMOLYZING AND SH-INHIBITING FACTORS

TOKURO SATO, TAEKO SUZUKI AND TOMITARO FUKUYAMA

*From the Department of Nutrition and Biochemistry, Institute of Publich Health Minato-ku, Tokyo, Japan*

Received for publication September 7, 1961

CIGARETTES for the study of chemical composition of smoke are usually conditioned at 60 per cent r.h. and 70° F. for at least 48 hours (Bentley and Burgan, 1959). But it is common to see people in Europe smoke very dry cigarettes in heated rooms in winter. A statistical study has shown that death rates due to lung cancer which occur in countries where principally cigarettes have been smoked may be correlated with the existence or absence of heating facilities which make cigarettes dry (Sato and Sakai, 1960; Sato *et al.*, 1961). In Japan the rate is very low and the influence on it of cigarette smoking is difficult to detect, although the consumption of cigarettes is at the same level as in Finland and Austria where the rates are very high.

In the present study cigarette smoke has been studied for the mode of adhesion and for SH reactive substances which inhibit SH enzymes in smoke solution, and a haemolyzing factor contained in water insoluble fraction of smoke condensate. These activities have been studied with special reference to the effects of cigarette moisture content.

### EXPERIMENTAL AND RESULTS

### *I. General conditions for the trapping of smoke*

Most of the smoke inhaled into the lung is seen to be exhaled. This means that only a small portion of the visible part of the smoke is retained in the body. During cigarette smoking the smoke is held for a short time in the mouth and then rapidly inhaled with a large volume of air into the lungs. This rapid inhalation makes it unlikely that the diluted smoke reaching the lungs has had time to become saturated with water vapour.

When smoke is forced through a capillary tube the amount of material which is deposited upon the capillary walls depends upon the rate of flow of the gas and upon the dimensions and shape of the capillary used. Accumulation of the smoke deposit tends to occur at bends or points of irregularity in a capillary.

Similarly, when smoke is bubbled through a liquid (buffer solution or organic solvent), the amount trapped in solution depends upon the rate of flow of gas.

### *II. Water insoluble fraction of cigarette condensate and its haemolyzing activity*

*Experiment IIa.*—Preparation of the water insoluble fraction of cigarette condensate.

The apparatus shown in Fig. 1 was used. Cigarette smoke (25 ml.) was forced from a dry syringe through the capillary tube (about 1·5 mm. internal diameter)

into 5 ml. of a buffer solution containing acetone in 0·5 to 1 second. The buffer solution was prepared by mixing a salt solution (aqueous sodium chloride (0·9 per cent w/v) : aqueous phosphate buffer (0·1 M, pH 7·4), 9 : 1) with an equal volume of acetone. The apparatus was maintained at 37° C. Any uncondensed smoke remaining in the capillary was displaced by 50 ml. of air. Smoke was taken at the rate of 1 two-second puff of 25 ml. per minute and 4 cm. of the cigarette was smoked (6 to 8 puffs). The capillary was removed from the apparatus and the inside walls were gently washed 10 times with buffer solution containing acetone and then washed 10 times with water.

FIG. 1.—Impinger for trapping smoke (see Experiment IIa in the text).

The quantity of smoke condensate prepared in this way depends upon the size and shape of the capillary tube used. In this work, variations due to this factor were obviated by the use of the same apparatus in all the experiments.

This fraction was soluble in acetone, ethanol, methanol and ethyl ether with yellow-brown colour. It did not migrate from ether solution to sodium hydroxide or hydrogen chloride solution. The acetone or alcohol solution became turbid on adding water.

This fraction did not react with glutathione or cysteine using the method which will be described later (Experiment IIId).

This fraction migrated with the solvent front on paper chromatograms run in butanol, acetic acid, water (4 : 1 : 1) and most of it did not move with 50 per cent alcohol.

*Experiment IIb.*—Haemolyzing activity of the water insoluble fraction.

A solution of the water insoluble fraction of smoke condensate was prepared by dissolving the material in 1 ml. acetone and adding 15 ml. of the sodium chloride-phosphate buffer solution used in the preparation (Experiment IIa).

A measured volume of this condensate solution was pipetted out and made up to 2 ml. with acetone/salt-buffer solution (1 : 15). The diluted solution was maintained at 40° C. and 0·1 ml. of pig red blood cells was added. The blood cells had been washed with sodium chloride solution (0·9 per cent w/v), adjusted to contain 0·5 mg./0·1 ml. of haemoglobin and stored in a refrigerator. The mixture was stirred occasionally and the haemolyzation time was recorded. The haemolyzing activity paralleled the intensity of yellow colour when dissolved in acetone, or the turbidity when mixed with the salt solution. The quantity of the condensate solution necessary to haemolyze the red blood cells just in 120 minutes was taken as a measure of the concentration of the agent in the solution (Table II).

Water insoluble fraction obtained in a solution devoid of acetone contained much of the haemolyzing agent, but lost most of it, when it was washed off with the solution containing acetone, leaving a small amount of residue.

*Experiment IIc.*—The quantity of water insoluble fraction according to cigarette moisture.

Water insoluble fraction of cigarette condensate was obtained according to the method described in Experiment IIa. The fraction, made by a dry cigarette at 20° C., 50 per cent r.h., was dissolved in 11 ml. of acetone and the concentration was measured by absorption at 400 m$\mu$. with 13 mm. light path. The extinction coefficient averaged 0·158 for 15 cases with a distribution range of $\pm$40 per cent. The concentration from moist cigarettes (13 per cent moisture) averaged 0·075 with the range of $\pm$ 50 per cent. The concentration of the tar before washing was independent of the moisture of cigarettes. The absorption curve of the fraction in methanol declined from 220 m$\mu$. to 450 m$\mu$. without any special absorption band.

Smoke of dry cigarettes from a wet syringe deposited half the quantity of the fraction as from a dry syringe. The fraction, from dry cigarettes smoked at the atmosphere of 37° C., and 15 per cent r.h. into a dry syringe and trapped at 20° C., showed the extinction of 0·25, but that from moist cigarettes at 37° C., 15 per cent r.h., showed the same level as smoked at room temperature (Table I).

TABLE I.—*Quantity of Water Insoluble Fraction (Experiment IIa) According to Cigarette Moisture and Smoking Conditions. The Fraction was Dissolved in 11 ml. of Acetone and Determined at 400 m$\mu$*

| Smoking condition | Moisture of cigarette | Number of cases | Mean value | Range of distribution (%) |
|---|---|---|---|---|
| 20° C., 60 ~ 70% r.h. | dry | 15 | 0·158 | $\pm$40 |
| | 13% | 15 | 0·075 | $\pm$50 |
| 37° C., 15% r.h. | dry | 8 | 0·250 | $\pm$50 |
| | 13% | 8 | 0·078 | $\pm$50 |

*Experiment IId.*—Effect of temperature and aeration on the haemolyzing activity.

The fraction was dissolved in the acetone and salt solution (Experiment IIb) and heated at 55° C. for 30 minutes. Haemolyzing activity decreased as shown in

Table II. There was an exceptional case which showed an increase when smoke was taken at the rate of successive four-second puffs of 50 ml. (Table II).

TABLE II.—*Effect of Temperature and Aeration on Haemolyzation. Method is Described in Experiment IIb in the Text*

Example

| | | | | | | | |
|---|---|---|---|---|---|---|---|
| Quantity of the solution (ml.) | 0·70 | 0·60 | 0·50 | 0·40 | 0·38 | 0·30 | 0·20 |
| Haemolyzation time (minutes) | 25 | 35 | 58 | 100 | 120 | 180 | none* |

* After 5 hours left overnight at 20° C.

| Condition of smoking | Treatment of the fraction | Quantity of the solution haemolyzing in 120 minutes |
|---|---|---|
| 25 ml./2 sec. suction in 1 min. interval: 4 cm. of a cigarette was smoked | Without treatment | 0·38 |
| | 55° C., 30 minutes | 0·48 |
| | 55° C., 30 minutes with aeration | 0·54 |
| 50 ml./4 sec.: successively smoked for 4 times | Without treatment | 0·38 |
| | 55° C., 30 minutes | 0·29 |
| | 55° C., 30 minutes with aeration | 0·50 |

When the solution was bubbled with air as well as heating at 55° C., the activity decreased more than when heated without aeration (Table II). The activity was not influenced by the pre-incubation of the fraction with cysteine (pH 7·4).

About one third of the activity was lost when the solution was left at 20° C. for 24 hours. The decrease in activity was minimised by keeping the solution frozen at −20° C. (Table III).

TABLE III.—*Preservation of the Water Insoluble Fraction and Haemolyzation (see Experiment IIb in the text)*

| Experiment number | Preserving method | Quantity of the solution haemolyzing in 120 minutes (ml.) |
|---|---|---|
| 1 | 0 time | 0·37 |
| | 24 hr at room temp. | 0·59 |
| | 24 hr. at 37° C. | 0·61 |
| | 24 hr. at −20° C. | 0·38 |
| | 48 hr. at room temp. | 0·64 |
| | 48 hr. at 37° C. | 0·73 |
| | 48 hr. at −20° C. | 0·38 |
| 2 | 0 time | 0·36 |
| | 24 hr. at room temp. | 0·53 |
| | 120 hr. at room temp. | 1·50 |
| 3 | 0 time | 0·80 |
| | 24 hr. at room temp. | 1·15 |
| | 47 hr. at room temp. | 1·55 |
| 4 | 0 time | 0·29 |
| | 24 hr. at room temp. | 0·48 |
| | 48 hr. at room temp. | 0·65 |
| | 72 hr. at room temp. | 0·86 |

*Experiment IIe.*—Quantity of condensate at a bending part of a capillary.

Trapping experiments were carried out using a capillary containing a bent portion near the exit. Smoke condensate from moist cigarettes tended to spread along the tube after being deposited at the bend. This spreading of the condensate only occurred after passing 50 ml. of air through the capillary in experiments with smoke from dry cigarettes.

The condensate which accumulated at the bend in the capillary was dissolved in 11 ml. of acetone and the light absorption was measured at 400 m$\mu$. with a 13 mm. path. The amount of smoke from dry digarettes condensing in the bent capillary was double that obtained from moist cigarettes (Table IV).

TABLE IV.—*Quantity of Condensate at a Bending Part of a Capillary*

| Speed of blowing smoke into the capillary | Moisture of cigarette | Number of cases | Mean value | Range of the distribution (%) |
|---|---|---|---|---|
| 50 ml./3~4 sec. | dry | 8 | 0·115 | ± 30 |
| | 13% | 8 | 0.054 | ± 30 |
| 50 ml./0·5~1·0 sec. | dry | 8 | 0·298 | ± 30 |
| | 13% | 8 | 0·153 | ± 30 |

Smoke was taken at the speed of 50 ml./4 sec. in 1 minute interval, 4 times for a dry cigarette, 5 times for a moist cigarette. The capillary was about 1·2 mm. in diameter (see Experiment IIe, in the text).

Using a narrow capillary (about 0·5 mm. diameter) and controlling the smoke flow so that the minimum of condensate was deposited from smoke of moist cigarettes, gave conditions which produced 3 times the amount of condensate when the experiment was repeated with smoke from dry cigarettes.

*III. SH binding substances and the inhibition of succinic dehydrogenase and urease*

*Experiment IIIa.*—Inhibition of succinic dehydrogenase.

Cigarette smoke was passed through 15 ml. of phosphate buffer (0·1 M, pH 7·4) at 37° C. through a pipette, the end of which was not so small as to avoid the accumulation of the smoke condensate, at the rate of 1 two-second puff of 25 ml. per minute for 4 cm. of a cigarette. Mitochondria of the heart of a pig were isolated (Bonner, 1955). The mitochondrial preparation (0·2 ~ 0·4 ml.) was incubated with the smoke solution (1 ~ 3 ml.). Succinic dehydrogenase activity was determined using methylene blue after 1·5 ~ 2·5 hours, and a marked decrease was observed. The water insoluble fraction of the condensate (Experiment IIa) did not inhibit the enzyme activity.

*Experiment IIIb.*—The effect of temperature and SH reagents on the inhibition of succinic dehydrogenase.

The smoke solution (4 ml.) was pre-incubated with cysteine or glutathione (1 mg., pH 7·4) at 37° C. for 30 minutes. The inhibition of succinic dehydrogenase by the smoke solution decreased practically to zero.

The smoke solution was heated at 100° C. for 10 minutes and the inhibition decreased, but it was difficult to seen any difference after aeration (Table V).

When the smoke solution was left overnight at 20° C., the inhibition decreased markedly, and the decrease for the first 24 hours was the largest. It was difficult to preserve the inhibition activity even at —20° C. (Table VII).

The succinic dehydrogenase inhibiting activity of the smoke solution from dry cigarettes smoked at 20° C. was often a little stronger than that from moist

TABLE V.—*Inhibition of Succinic Dehydrogenase by Smoke Solution and the Effect of Temperature (see Experiment III in the Text)*

| Sample number | Treatment of smoke solution | Succinic dehydrogenase activity with the pre-incubation of mitochondria with smoke solution for 1·5 hr. | 2·5 hr. |
|---|---|---|---|
| 1 | Without treatment | 16·5 min. | 36 min. |
| | 100° C. for 10 min. | 10·5 | 17 |
| | Without smoke solution | 6·5 | 6·5 |
| 2 | Without treatment | 11·5 min. | 30 min. |
| | 100° C. for 10 min. | 5·7 | 11 |
| | Without smoke solution | 3·4 | 3·5 |
| 3 | Without treatment | 11·5 min. | 40 min. |
| | 100° C. for 10 min. | 5·7 | 10·5 |
| | Without smoke solution | 3·4 | 3·5 |

2·0 ml. of smoke solution and 0·3 ml. of mitochondira solution was added to 3·7 ml. of phosphate buffer solution (0·1 M, pH 7·4). After the pre-incubation 2·5 ml. was taken out of it and the succinic dehydrogenase activity was determined using methylene blue.

TABLE VI.—*Quantity of Smoke Solution and the Inhibition of Succinic Dehydrogenase. Pre-incubation was made with* 0·3 *ml. of Mitochondria and Phosphate Buffer Solution : Total Volume* 6 *ml. (see Table V)*

| Quantity of smoke solution | Succinic dehydrogenase activity with pre-incubation of 1·5 hours |
|---|---|
| 5 ml. | 39 min. |
| 2·5 ml. | 25 |
| 1·25 ml. | 11 |
| 0·625 ml. | 6 |
| Without smoke solution | 4·5 |

TABLE VII.—*Preservation of Smoke Solution and Succinic Dehydrogenase Inhibiting Activity (see Table V)*

| Experimental number | Preserving method | Succinic dehydrogenase activity (minutes) 1·5 hr. pre-incubation | 2·5 hr. pre-incubation |
|---|---|---|---|
| 1 | 0 time | 16·0 | 24·0 |
| | 24 hr. at 37° C. | 9·5 | 9·5 |
| | 24 hr. at −20° C. | 11·5 | 12·0 |
| | 47 hr. at 37° C. | 8·0 | 8·5 |
| | 48 hr. at −20° C. | 12·0 | 12·5 |
| | Without smoke solution | 7·0 | 7·0 |
| 2 | 0 time | 14·0 | 18·0 |
| | 24 hr. at 37° C. | 9·5 | 9·3 |
| | 24 hr. at −20° C. | 11·5 | 11·5 |
| | 48 hr. at 37° C. | 8·5 | 8·5 |
| | 48 hr. at −20° C. | 12·0 | 12·5 |
| | Without smoke solution | 6·0 | 6·0 |

cigarettes ; this effect did not appear to be significant. The haemolyzing activity of the smoke solution was stronger by 30 per cent in the solution from dry cigarettes than in that from moist cigarettes.

*Experiment IIIc.*—Inhibition of urease by smoke solution.

A solution of crystalline urease (0·7 mg. ammonium production from urea in 5 minutes) was pre-incubated with the smoke solution obtained as described in

Experiment IIIa, and the activity of the urease was tested (Sumner, 1955). A small portion of the reaction mixture was taken out 5 minutes after the addition of urea, diluted with water, and the ammonia was detected with Nessler reagent. Almost complete inhibition was observed using 10 ml. of the smoke solution with 3 hours' pre-incubation.

The smoke solution (4 ml.) was pre-incubated with SH reagents (1 mg., pH 7·4 solution of cysteine or glutathione) at 37° C. for 30 minutes. The inhibition of urease by the smoke solution was almost completely antagonized by this procedure. On heating the smoke solution at 100° C. for 20 minutes the inhibition did not decrease. But when the solution was left overnight at 20° C., the inhibiting activity decreased considerably.

*Experiment IIId.*—Detection of SH combined substances on paper chromatograms.

Smoke solution was obtained by the method of Experiment IIIa, and 10 ml. was used for the test. Glutathione or cysteine (1 $\sim$ 2 mg., pH 7·4) was added to the solution and incubated at 37° C. for 15 minutes. The combined substances were adsorbed to charcoal and eluted with ammonia/methanol. The eluate was applied to paper and chromatographed with the solvent of butanol, acetic acid, water (12 : 3 : 5) by ascending method. Completed chromatograms were examined for colour, fluorescence under ultraviolet light, ninhydrin reaction and organic sulphur test by bichromate solution spray followed by silver nitrate solution (Booth *et al.*, 1960). Seven spots were detected on the paper, but details of them await further elucidation (Table VIII).

TABLE VIII.—*Glutathione Combined Substances in Smoke Solution (see Experiment IIId in the Text). Solvent: Butanol, Acetic Acid, Water* (12 : 3 : 5) *by Ascending Method*

| | Number of spot | | | | | | |
|---|---|---|---|---|---|---|---|
| | 1 | 2 | 3 | 4 | 5 | 6 | 7 |
| Rf value | 0·04 | 0·23 | 0·30 | 0·42 | 0·55 | .. | .. |
| | .. | 0·20 | 0·27 | 0·38 | 0·48 | 0·63 | 0·75 |
| | | $\sim$0·25 | $\sim$0·33 | $\sim$0·45 | $\sim$0·62 | $\sim$0·70 | $\sim$0·85 |
| Colour | yellow | yellow | .. | .. | .. | .. | .. |
| | orange | orange | | | | brown | brown |
| Fluorescence | .. | + | + | .. | absorption | .. | .. |
| Organic sulphur test | +++ | +++ | ++ | + | ++ | ++ | ++ |
| Ninhydrin R. | + | +++ | + | + | ++ | − | − |

Ninhydrin reaction : No. 2 is clear, several patterns appear around No. 1 ; No. 3 and No. 4 are faint and diffuse. Smoke solution from dried cigarettes contains much of No. 1 and No. 5.

The combination was accelerated by the addition of the supernatant liquid from liver homogenates of the rat as has been observed in the case of the epoxide of dihydronaphthalene (Booth *et al.*, 1960).

The water insoluble fraction of the condensate in the salt solution devoid of acetone (Experiment IIb) contained SH binding substances as well as the haemolyzing factor.

Substances No. 1 and No. 5 (Table VIII) formed from the condensate from dried cigarettes appeared in about double the quantity as from the condensate from moist cigarettes. Leaving the smoke solution at 37° C. for four days caused the spots below No. 4 to diminish markedly. Heating the solution at 100° C for 10 minutes caused the spots below No. 4 to decrease and spots No. 6 and No. 7 appeared to increase with this treatment.

### DISCUSSION

In the present study it has been demonstrated that the water insoluble fraction of the condensate of cigarette smoke possesses haemolytic properties, and the buffer soluble fraction contained SH binding and SH enzyme inhibiting substances. Cigarette smoke has been shown to precipitate at bends or points of swelling in a capillary. A reduction in the diameter of a capillary, or an increase in the velocity of smoke forced through it, results in the precipitation of a greater quantity of condensate on the walls. Smoke from dry cigarettes precipitates more effectively on these parts of a capillary than that from moist cigarettes. The precipitate from dry cigarettes has been shown to be resistant to washing with the mixture of acetone and salt solutions.

Without the haemolyzing activity of the precipitate, the cell membrane of the respiratory system will remain intact and the SH inhibiting and combining substances will be unable to react with the cell constituents inside the membrane. The results presented in this work suggest that smoke of dry cigarettes will condense on the respiratory walls in greater quantity and will remain longer at bending or swelling parts of capillaries (physiological and pathological) with the result of greater injury to the wall cells than the smoke of moist cigarettes. Repeated injuries of cells might lead to malignant change as discussed by Smithers (1960). Also the existence of SH combining substances in the smoke solution suggests that some of them might have radiomimetic action. At the present stage of study, it is difficult to evaluate the results obtained in this paper with respect to carcinogenesis. However, the level of moisture in cigarettes and the mode of precipitation of cigarette smoke are conditions which do not seem to have been taken into consideration hitherto.

Any theory proposed for the causation of lung cancer should be able to explain the high incidences of the cancer in England, Austria and Finland and the very low incidence in Japan. In these countries cigarettes principally have been smoked at similar levels. In winter, dry cigarettes have been observed to be common in the European countries but seldom in Japan. The different heating facilities existing in these countries may offer a key to the problem.

There may be many other factors which should be studies in connection with the moisture content of cigarettes, such as electrical charge, of smoke from dry cigarettes together with its pathogenic effects on the walls of the respiratory system. Although some factors have been considered in the present study, further studies appear to be needed for a most complete elucidation of the problem.

The observations that haemolyzing and SH binding activities of smoke condensate are rather unstable show that it is advisable to use freshly prepared smoke condensate for the experimental cancer study of cigarette smoke.

### SUMMARY

Cigarette smoke was found to precipitate at the bending or swelling portions of a capillary. The extent of this precipitation depends on the velocity of the smoke and narrowness of the capillary.

Water insoluble but organic solvent soluble fraction of smoke condensate haemolyzed red blood cells effectively. This activity decreased by one third in 24 hours at room temperature and was susceptible to aeration but not to SH reagents.

Smoke trapped in phosphate buffer solution was found to contain several SH binding substances and to inhibit SH enzymes such as succinic dehydrogenase and urease. This activity decreased markedly in 24 hours at room temperature and was susceptible to SH reagents.

Smoke from dry cigarettes has been shown to be more easily precipitated than smoke from moist cigarettes and the precipitate is more resistant to removal. From these observations it appears that smoke from dry cigarettes acts first to injure the cell membrane and allows SH binding substances to enter inside the cell membrane more effectively than smoke from moist cigarettes.

We are indebted to Professor A. Haddow and Professor E. Boyland of the Chester Beatty Research Institute, London, for their kind interest, and to Dr. P. Sims of the Institute for teaching methods and sharing chemicals for the experiment.

### REFERENCES

BENTLEY, H. F. AND BURGAN, J. G.—(1959) ‘ Cigarette Smoke Condensate ’, Tobacco Manufacturers Standing Committee, Research Paper No. 4, London.

BONNER, W. D.—(1955) ‘ Methods in Enzymology ’, Vol. 1, p. 722. New York (Academic Press).

BOOTH, J., BOYLAND, E., SATO, T. AND SIMS, P.—(1960) *Biochem. J.*, **77**, 182.

SATO, T. AND SAKAI, Y.—(1960) *Sogo Igaku* (*General Medicine*), **17**, 817.

*Idem*, FUKUYAMA, T., SUZUKI, T., TAKAYANAGI, J. AND SAKAI, Y.—(1961) *Bull. Inst. Publ. Hlth.* **10**, 31.

SMITHERS, D. W.—(1960) ‘ A Clinical Prospect of the Cancer Problem ’. Edinburgh (Livingstone), pp. 76 and 96.

SUMNER, J. B.—(1955) ‘ Methods in Enzymology ’, Vol. 2, p. 378. New York (Academic Press).